# Function of formyl peptide receptor 2 in adriamycin resistance of breast cancer

**DOI:** 10.3389/ebm.2024.10281

**Published:** 2025-01-15

**Authors:** Landi Su, Jingjing Li, Li Qin, Yang Feng, Dingwen Xu

**Affiliations:** School of Medicine, Yangzhou Polytechnic College, Yangzhou, Jiangsu, China

**Keywords:** FPRL2, breast cancer, adriamycin resistance, apoptosis, molecular docking

## Abstract

FPRL2 has been shown to be associated with a variety of tumours but has not been well studied in breast cancer. In this study, We combine molecular biology techniques with bioinformatics to analyze the role of FPRL2 in breast cancer and adriamycin resistance. By utilizing bioinformatics, we mine TCGA and GEO public databases to assess FPRL2 expression in breast cancer patients and its correlation with patient prognosis. Additionally, we employ the DepMap tool to probe the CCLE database, examining the relationship between FPRL2 gene effects and adriamycin sensitivity. Chemosensitivity of Adriamycin in breast cancer cells was tested by CCK-8 method. The apoptosis of breast cancer cells was determined by flow cytometry assay. Expression of p-ERK5 and p-AKT was determined by Western blot assay. Our results indicate that the expression level of FPRL2 in tumor tissues of breast cancer patients is significantly higher than that in normal tissues, and it correlates with poor prognosis in patients. Furthermore, the expression level of FPRL2 in tumor tissues of adriamycin-resistant breast cancer patients is also significantly higher than that in adriamycin-sensitive patients. The IC_50_ (Inhibitory Concentration 50). Of Adriamycin was significantly lower in FPRL2 silenced cells than those control cells. The apoptosis was markedly increased in FPRL2-silenced cells. p-ERK5 and p-AKT in breast cancer cells was significantly reduced after FPRL2 knocked down. In Conclusion, FPRL2 mediates Adriamycin resistance in breast cancer cells, and knockdown of FPRL2 increased apoptosis and decreased Adriamycin resistance in breast cancer cells.

## Impact statement

Our results demonstrate for the first time that FPRL2 is highly expressed in breast cancer and adriamycin-resistant breast cancer cells and that knockdown of FPRL2 increases adriamycin-induced apoptosis in breast cancer cells. Our results provided clues for overcoming adriamycin resistance in breast cancer.

## Introduction

Breast cancer ranks as the most prevalent cancer type among women. Recent data indicates approximately 2.3 million new cases globally. In 112 countries, breast cancer serves as a leading cause of cancer-related deaths. Furthermore, estimates suggest that by 2040, this figure will surpass 3 million cases [1]. Breast cancer surgery plus chemotherapy is still the primary means of treatment for breast cancer. Surgical treatment of breast cancer has made significant progress, and minimally invasive treatment of breast cancer has dramatically improved the quality of life of patients without affecting the efficacy of surgery [2]. However, resistance to chemotherapy drugs is still a significant cause of breast cancer recurrence, metastasis, and death [3, 4].

The family of G protein-coupled receptors (GPCRs) is involved in a variety of physiological functions, including tumor growth and metastasis, and aberrantly expressed or aberrantly activated GPCRs are involved in various aspects of cancer progression such as tumor growth, invasion, migration, survival, and metastasis [5], which has led to the emergence of GPCRs as an essential target for tumor drug resistance [6]. Formyl peptide receptors (FPRs) are cell surface pattern recognition receptors (PRRs) that belong to the evolutionarily conserved family of G protein-coupled receptors (GPCRs). The formyl peptide receptor family consists of the Formyl peptide receptor (FPR), the Formyl peptide receptor-like 1 (FPRL1), and the Formyl peptide receptor-like 2 (FPRL2). Formyl peptide receptors are highly expressed in phagocytic leukocytes, and activation of the receptors by agonists triggers a series of signaling events that result in leukocyte activation, cell chemotaxis, phagocytosis, release of inflammatory mediators, and other biological effects, thus playing an essential role in the host defense response against pathogen infection [7]. The distribution of the formyl peptide receptor is not limited to phagocytosis of leukocytes but is also expressed in tumor cells such as colon cancer, which enhances drug resistance, and knockdown of FPR2 reduces the tumorigenicity of colon cancer [8, 9]. However, its expression on breast cancer tissues and its biological role have been little studied. This study investigates the expression of FPRL2 in breast cancer and explores the relationship between FPRL2 and doxorubicin resistance in breast cancer.

## Materials and methods

### Materials

#### Cell lines

The human breast adenocarcinoma resistant to Adriamycin cell line MCF-7/ADM and human adenocarcinoma cell line MCF-7 were purchased from the Shanghai Institute of Biochemistry and Cell Technology, Chinese Academy of Sciences.

#### Reagents and consumables

Adriamycin for injection was purchased from Shanghai Yuan Ye Company; DMEM medium and fetal bovine serum were purchased from Gibco, USA; RNA extraction kit was purchased from Qiagen, United States; CCK-8 (cell counting kit-8) kit was purchased from Tongrentang, Japan; Lipofectamine™ RNAiMAX was purchased from Thermo Electron, United States; RevertAidTM First Strand cDNA Synthesis Kit was purchased from Fermentas, Canada; Realtime PCR Kit ABI SYBR Green Master Mix was purchased from Invitrogen, United States; Realtime PCR Kit ABI SYBR Green Master Mix was purchased from Invitrogen, United States; Reverse Transcription RevertAidTM First Strand cDNA Synthesis Kit was purchased from Fermentas, Canada; Real-time PCR kit ABI SYBR Green Master Mix was purchased from Invitrogen, United States; RNase and propidium iodide (PI) were purchased from Sigma; rabbit anti-AKT, p-AKT and GAPDH were purchased from Cell Signaling, United States; rabbit anti-FPRL2 antibody, FPRL2 siRNA and its control siRNA, and HRP-labeled goat-anti-rabbit IgG were purchased from Santa Cruz, United States; immunohistochemistry assay SuperPictureTM 3rd Gen IHC Detection kit was purchased from Invitrogen, United States. The IHC Detection kit was purchased from Invitrogen, United States.

### Methodology

#### Bioinformatics analysis

TCGA breast cancer data and METABRIC breast cancer data were downloaded by cBioportal, and then FPRL2 expression was analyzed. The correlation between FPRL2 and the prognosis of breast cancer patients was analyzed by KM-plotter^1^. The correlation of gene effects with drug sensitivity in CCLE (Cancer Cell Line Encyclopedia) database was analyzed using the DepMap platform^2^. Molecular docking was performed by AutodockVina 1.2.2^3^ to analyze protein-molecule binding energy and interaction patterns.

#### Cell culture and siRNA transfection

The above cell lines were routinely cultured in DMEM medium containing 10% fetal bovine serum and dual antibodies at 37°C, 5% CO_2_, and 1 μg/mL of Adriamycin was added to the medium of MCF-7/AMD to maintain its resistance. To knock down endogenous FPRL2, cells were transiently transfected with 100 nM mouse siRNA targeting FPRL2 (si-FPRL2) or non-silencing control siRNA (si-NC) in the antimicrobial-free medium using Lipofectamine™ RNAiMAX in both cell lines.

#### Real-time PCR

After the above cell lines were routinely cultured, RNA extraction and reverse transcription were performed. PCR reactions were performed according to the instructions, respectively, with 5′-ACT​ACT​ACG​CCA​AGG​AGG​TCA​C-3′ as the upstream primer, 5′-GAG​CAA​CAC​GGG​GTT​CAG​GT-3 ′ as the downstream primer to amplify the mRNA of FPRL2, 5′- TGC​ACC​ACC​AAC​TGC​TTA​GC-3′ as the upstream primer, 5′- GGC​ATG​GGA​CTG​TGG​TCA​TGA​G-3 ′ as downstream primer amplified GAPDH mRNA as internal reference.2^−ΔΔCT^ method was used to process the amplified data.

#### IC50 of adriamycin measurements

Cell Counting Kit-8 was performed according to the instructions of Cell Counting Kit-8. PRRL2 knockdown and non-knockdown cell lines were planted in 96-well plates (100 μL, 2 × 10^3^/well) and treated with 0, 5, 10, 20, and 40 μg/mL of adriamycin (5 replicate wells per group) for 72 h. After that, the cell lines were added to CCK-8 solution and incubated at 37°C for 3 hours. The cell lines’ OD values were determined using an enzyme marker (Bio-Tek, Elx800, United States) at 450 nm. The half-maximal inhibitory concentration of the drug (IC_50_) was calculated from the absorbance report.

#### Western blot

First, treat the cells with an LC50 dose of doxorubicin for 24 h to obtain standard cell lysates. Next, perform SDS-PAGE electrophoresis followed by membrane transfer. After the transfer, incubate the membrane overnight at 4°C with primary antibodies (FPRL2, p-ERK5, p-AKT, GAPDH). The following day, wash the membrane three times with PBST, then incubate it at room temperature for 2 h with HRP-conjugated sheep anti-rabbit secondary antibodies. Finally, detect protein bands using ECL chemiluminescence.

#### Flow cytometry

Cells (5 × 10^6^ cells/tube) from various treatments were washed in cold PBS and fixed in 70% ethanol at 4°C. After PBS washing, RNase (500 U/mL) was treated at 37°C for 15 min, and DNA was stained with 50 μg/mL propidium iodide (PI) (dissolved in PBS). Apoptosis analysis was performed using a Becton Dickinson flow cytometer (BD-FACS Aria Ⅱ, California, United States) and the included software. Approximately 15,000 cells were counted per assay.

#### Statistical methods

GraphPad Prism 10 was used for statistical analysis of the data. The unpaired t-test was applied for comparisons between two groups of data that followed a normal distribution. For data that did not follow a normal distribution, the rank-sum test was used. A significance level of p < 0.05 was set as the criterion for significant differences.

## Results

### Bioinformatics analysis of FPRL2 expression and prognosis in breast cancer

**FIGURE 1:**
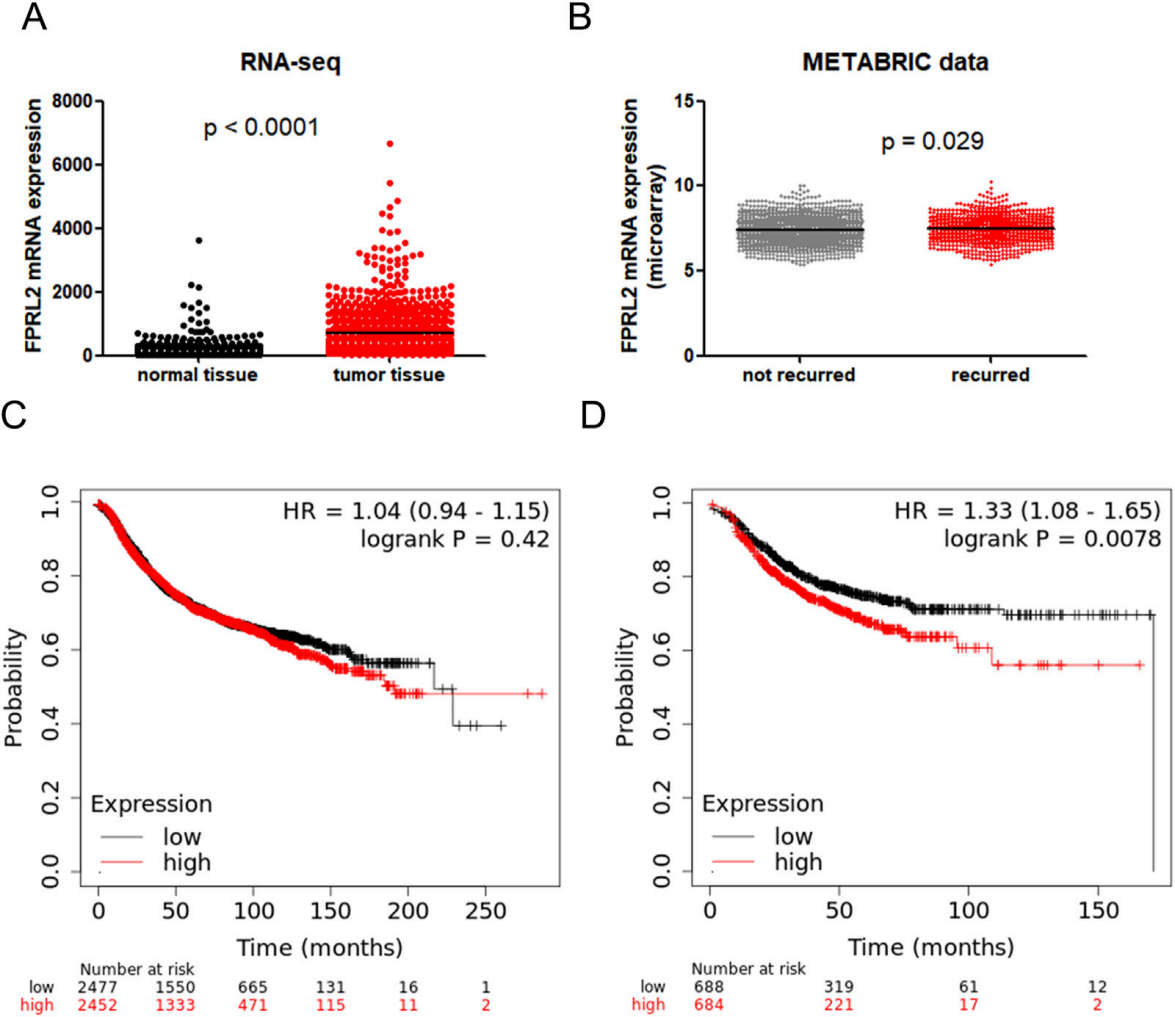
Bioinformatics analysis of FPRL2 expression and prognosis in breast cancer. **(A)** Data in TCGA and GTex breast cancer patients show that FPR3 is elevated in breast cancer. **(B)** The METABRIC database shows that FPR3 expression is higher in patients with recurrent breast cancer. **(C)** Correlation of FPR3 expression with disease progression-free survival in all breast cancers. **(D)** Correlation of FPR3 expression with disease progression-free survival in breast cancer treated with chemotherapy.

Through the TCGA database and METABRIC database, we found that FPRL2 expression was significantly elevated in breast cancer (P < 0.05) (Figure 1A), and the expression was higher in recurrent breast cancer than in non-recurrent breast cancer tissues (P < 0.05) (Figure 1B). By KM-plotter analysis, FPRL2 expression was found to be not significantly correlated with disease progression-free survival in breast cancer patients (P > 0.05) (Figure 1C). Interestingly, it was significantly correlated with disease progression-free rate in patients treated with chemotherapy (P < 0.05) (Figure 1D). This suggests that FPRL2 may affect the prognosis of patients by influencing the effect of chemotherapy.

### Expression analysis of FPRL2 in adriamycin-resistant breast cancer

**FIGURE 2:**
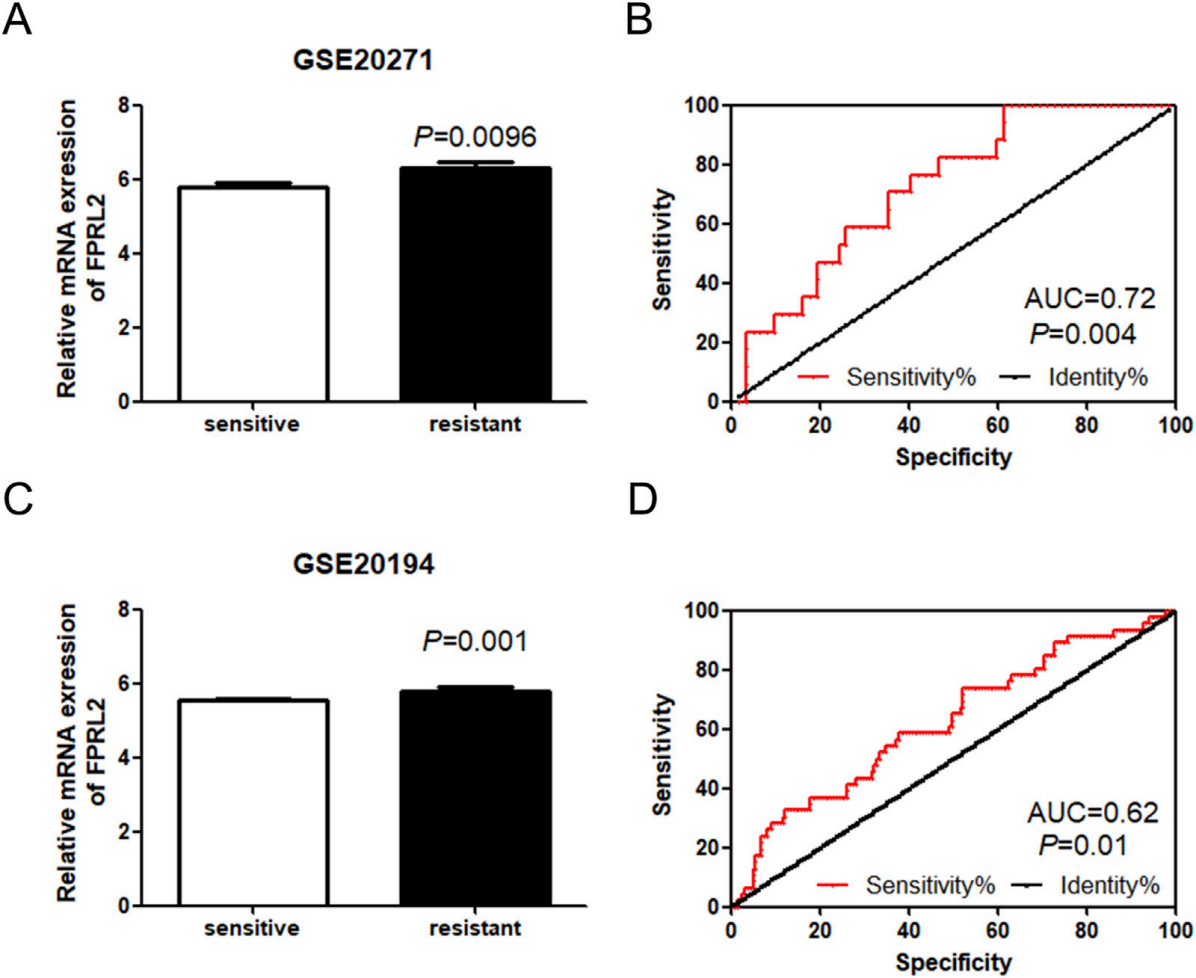
Expression analysis of FPRL2 in Adriamycin-resistant breast cancer. **(A, B)**: Expression of FPRL2 in Adriamycin-sensitive and drug-resistant **(A, B)**: Expression of FPRL2 in Adriamycin-sensitive and drug-resistant breast cancer samples in GSE20271 sample **(A)** and ROC curve analysis **(B)**. **(C, D)**: Expression of FPRL2 in Adriamycin-sensitive and drug-resistant breast cancer samples in GSE20194 sample **(C)** and ROC curve analysis **(D)**.

FPRL2 was significantly elevated (P < 0.05) in the GEO datasets GSE20271 and GSE20194, which were resistant to Adriamycin chemotherapy (Figures 2A, C). The ROC curve analysis revealed that FPRL2 could be used as an indicator for determining Adriamycin resistance in breast cancer (P < 0.05) (Figures 2B, D).

### Correlation between the FPRL2 gene effect and breast cancer cell adriamycin sensitivity

**FIGURE 3:**
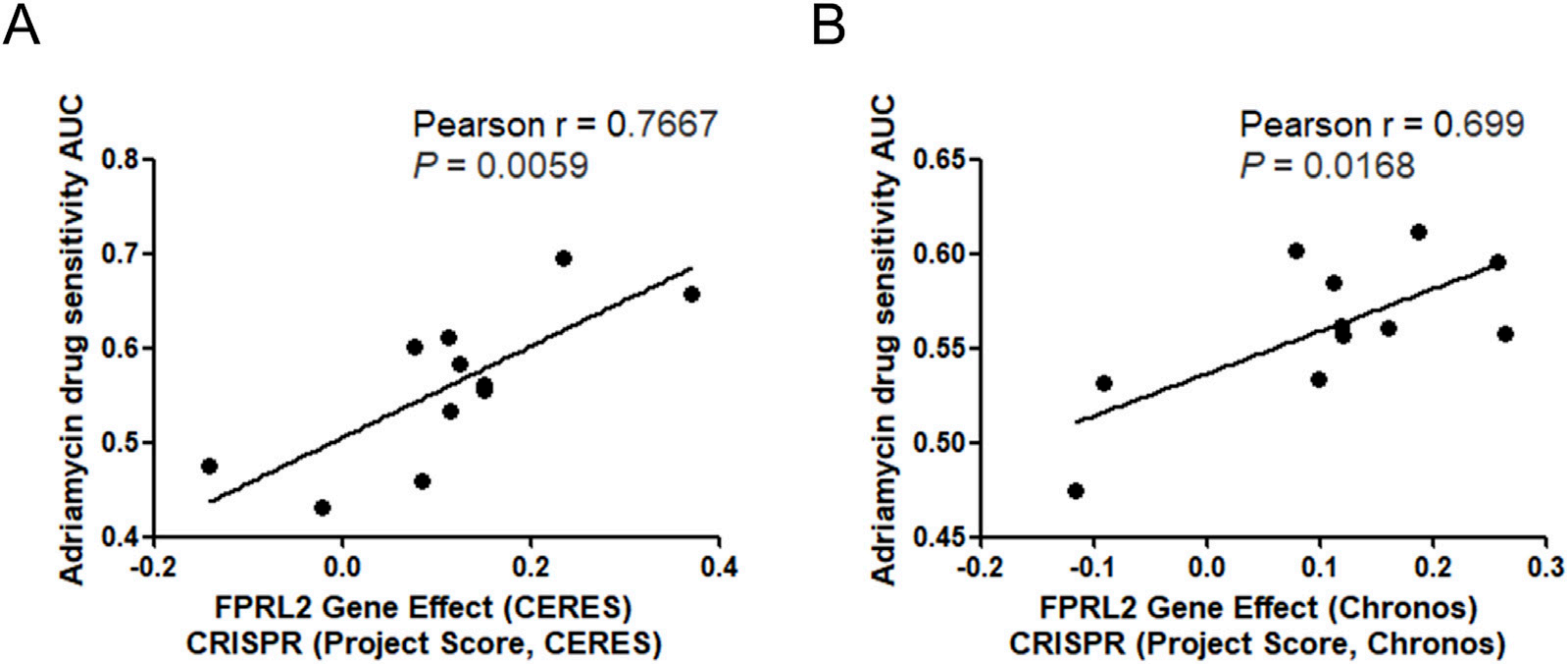
FPRL2 gene effect and Adriamycin sensitivity analysis. **(A)** FPRL2 gene effect and Adriamycin sensitivity analysis of breast cancer cells in CERES model. **(B)** FPRL2 gene effect and Adriamycin sensitivity analysis of breast cancer cells in Chronos model.

Analysis of the FPRL2 gene effect and breast cancer cell Adriamycin sensitivity by DepMap revealed a positive correlation between the FPRL2 gene effect and breast cancer cell Adriamycin sensitivity in two computational models, CERES and Chronos (Figures 3A, B). This indicates that the dependence on FPRL2 increases in Adriamycin-resistant breast cancer tissues.

### Molecular docking to validate the interaction of FPRL2 with adriamycin

**FIGURE 4:**
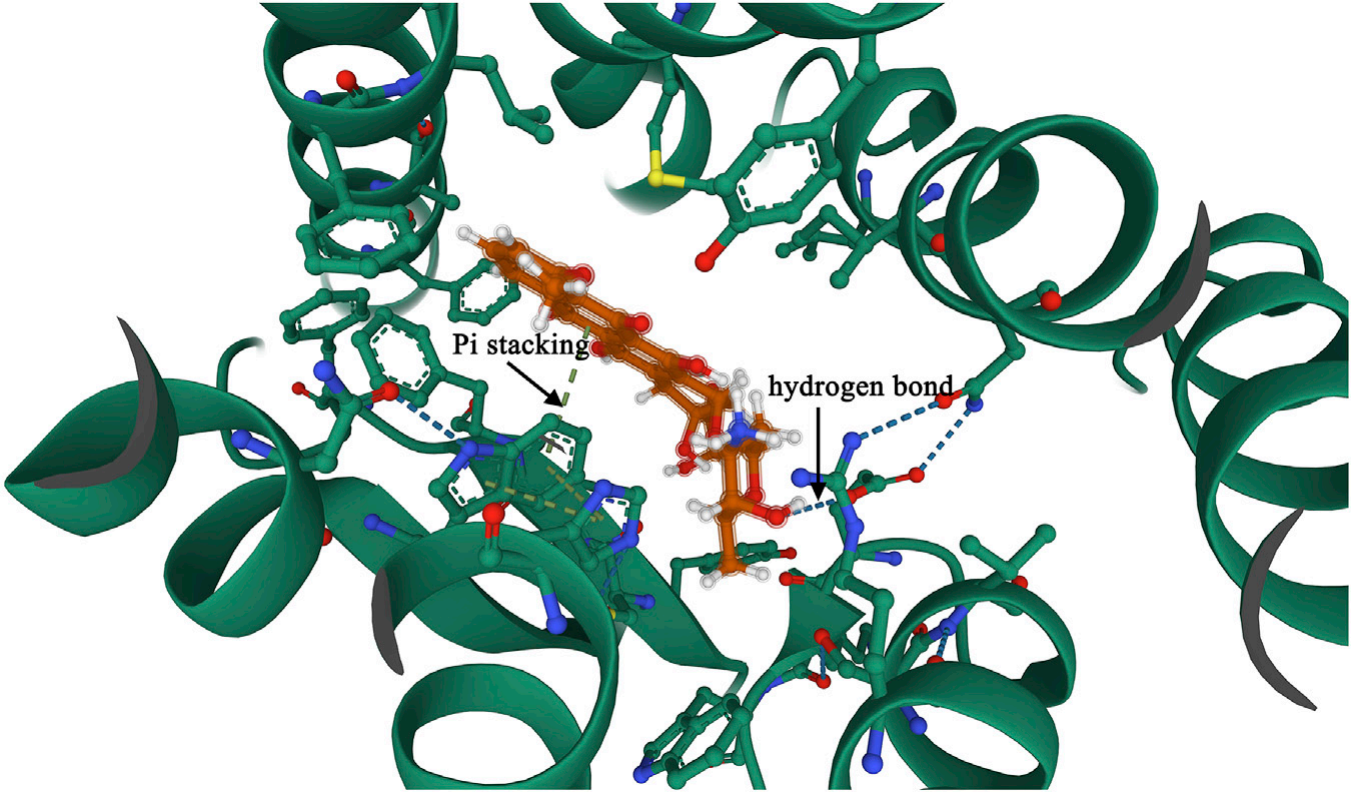
Molecular docking to verify the interaction of FPRL2 with Adriamycin. Note: FPRL2 protein in red and Adriamycin in green. Dashed lines are hydrogen bonding connections.

The molecular docking results showed that the affinity energy of Adriamycin to FPRL2 was −7.928 kcal/mol and that there were hydrogen bonding connections (hydrogen bonding) between Adriamycin and phenylalanine at position 5 (Phe 5), phenylalanine at position 178 (Phe 178), and aspartate at position 179 (Asn 179) of FPRL2 (indicated by arrows in Figure 4). bonds) (indicated by arrows in Figure 4). This implies that Adriamycin interacts with FPRL2.

### FPRL2 expression in breast cancer cell lines

**FIGURE 5:**
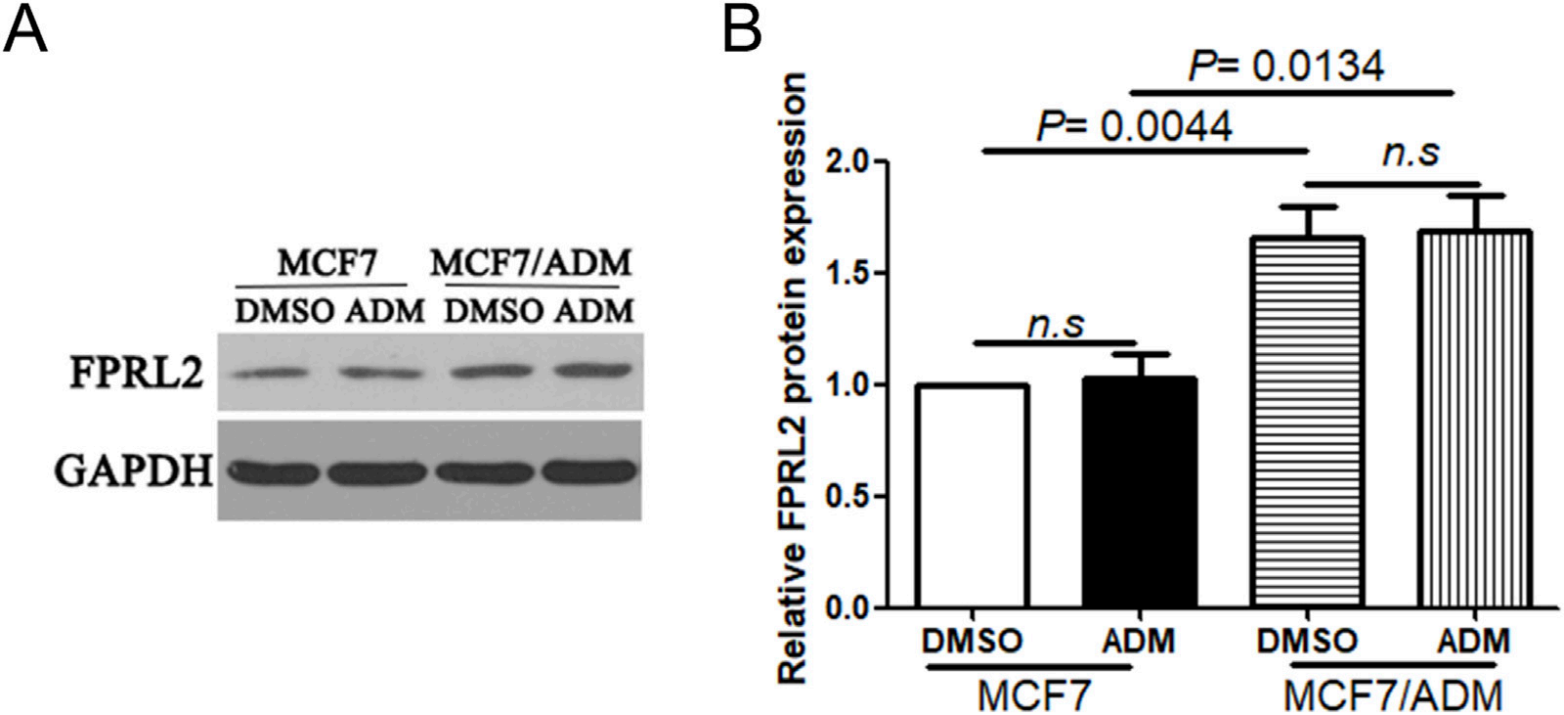
FPRL2 protein expression in breast cancer cell lines. **(A)** Protein expression of FPRL2 in Adriamycin-sensitive and drug-resistant cell lines of breast cancer tumors. **(B)** WB quantitative analysis.

The results of Western Blot showed that FPRL2 was significantly higher in resistant MCF-7 cells (MCF7/ADM) than in the sensitive group (MCF7) (P< 0.05). In addition, Adriamycin treatment did not affect the protein expression of FPRL2 in both MCF-7 and MCF7/ADM tumor cells (Figure 5).

### IC_50_ of Adriamycin and Adriamycin-induced apoptosis of breast cancer cells after FPRL2 knockdown

**FIGURE 6:**
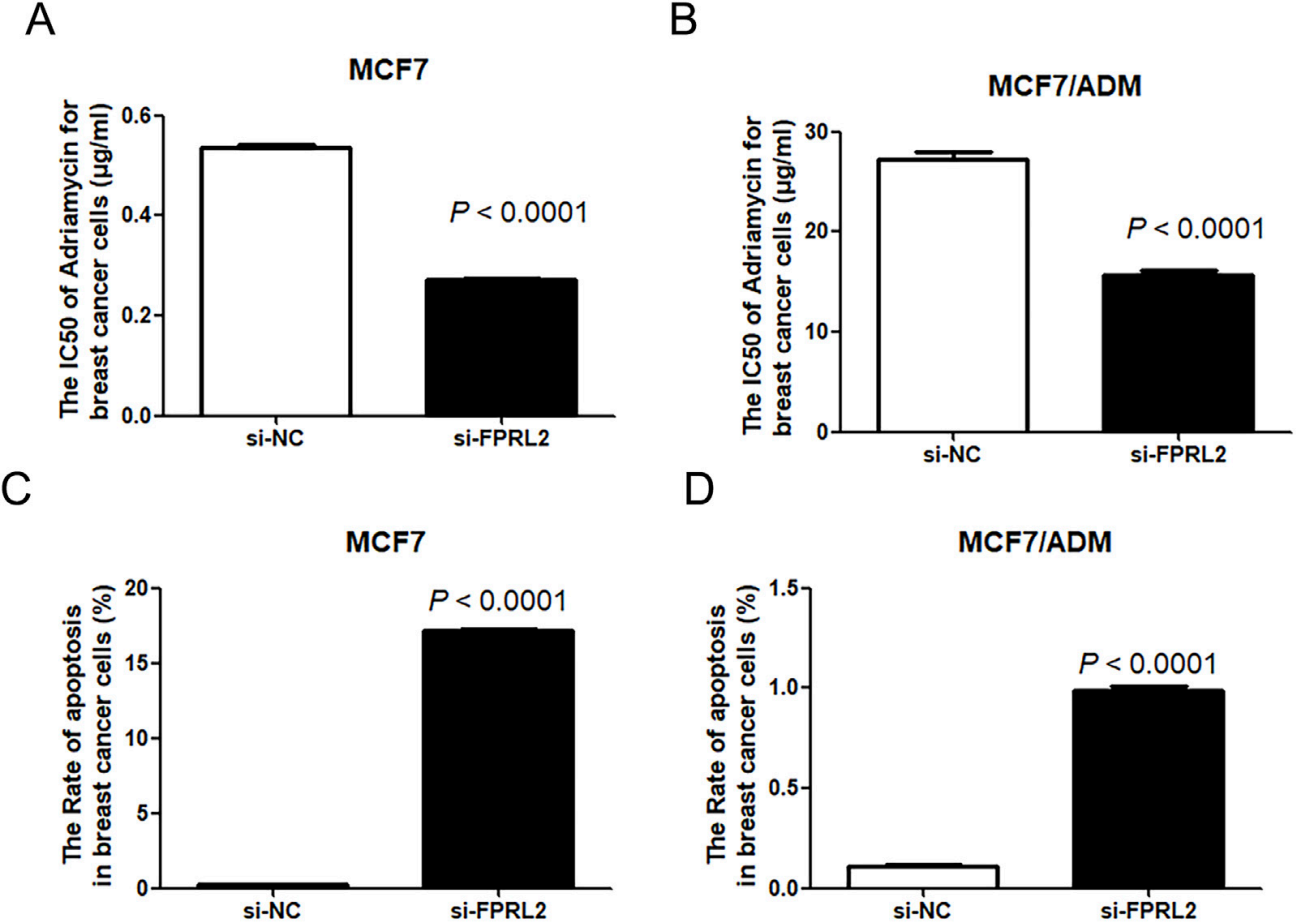
IC50 of Adriamycin and Adriamycin-induced apoptosis of breast cancer cells after FPRL2 knockdown. **(A)** The IC50 of Adriamycin in MCF7 after FPRL2 knockdown. **(B)** The IC50 of Adriamycin in MCF7/ADM after FPRL2 knockdown. **(C)** The Adriamycin-induced apoptosis rate of MCF7 after FPRL2 knockdown. **(D)** The Adriamycin-induced apoptosis rate of MCF7/AMD after FPRL2 knockdown.

**FIGURE 7:**
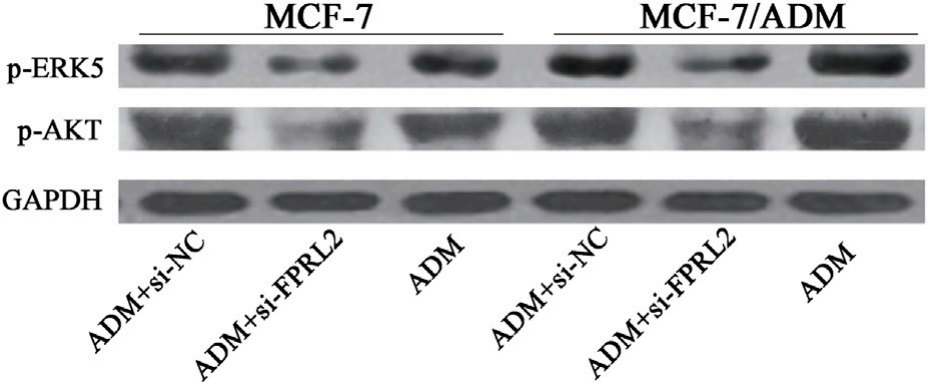
Expression of p-AKT and p-ERK5 in Adriamycin-treated breast cancer cells after FPRL2 knockdown.

As shown in Figure 7, the IC_50_ of Adriamycin on breast cancer cells after FPRL2 knockdown was significantly lower than that of the non-knockdown, both in resistant and non-resistant MCF-7 cells (P < 0.0001) (Figures 6A, B). In the same time, flow cytometry results showed that Adriamycin-induced apoptosis in breast cancer cells was significantly increased in both MCF7 and MCF7/ADM cell lines after FPRL2 knockdown (P < 0.0001) (Figures 6C, D).

### Expression of p-AKT and p-ERK5 in Adriamycin-treated breast cancer cells after FPRL2 knockdown

Western blot results showed that the expression levels of p-AKT and p-ERK5 in Adriamycin-treated breast cancer cells were lower after FPRL2 knockdown, in both MCF-7 and MCF-7/ADM (Figure 7).

## Discussion

Breast cancer is one of the common female malignant tumors, and the incidence and deaths have been increasing significantly in recent years [1]. In-depth research on breast cancer drug resistance can help to find ways to fight this common tumor. In this study, we reported for the first time that FPRL2 mediates Adriamycin resistance in breast cancer using public database mining, and breast cancer cell lines and utilizing a combination of bioinformatics and molecular biology experimental techniques.

Three subtypes of the formyl peptide receptor family are highly expressed in inflammatory cells, including FPR, FPRLl, and FPRL2. At the amino acid level, FPRLl shares 69% homology with FPR, and FPRL2 shares 56% and 83% homology with FPR and FPRLI, respectively. Numerous agonists of the formyl peptide receptor family of receptors include pathogen-derived, synthetic peptides, and endogenous substances from the host [10, 11]. Currently, the FPR family serves as an important pharmacological target for treating many inflammation-related diseases such as inflammatory lung disease, ischemia-reperfusion injury, neuroinflammation, and cancer [12–14]. Formyl peptide receptors are also expressed in breast cancer and are biomarkers for targeted therapies in the immune microenvironment of breast cancer [15]. The present study confirms the expression of FPRL2, a member of the formyl peptide receptor family, in breast cancer tissues and cell lines.

The formyl peptide receptor is a transmembrane G protein-coupled receptor that, upon activation, causes Ca^2+^ endocytosis and mediates cellular responses. It has been reported in the literature that the activation of the formyl peptide receptor mainly activates the ERK, AKT, and STAT signaling pathways, among which the ERK and AKT pathways are closely related to apoptosis and tumor cell resistance. The activation of the ERK signaling pathway leads to doxorubicin resistance in a breast cancer nude mouse model [16]. L-amino acid oxidase enhances the cytotoxicity of doxorubicin against breast and pancreatic cancer cells by attenuating ERK and AKT activities [17]. Ivermectin binds to the extracellular domain of EGFR, inhibiting the activation of EGFR and its downstream signaling cascade ERK/Akt/NF-κB, thereby enhancing the antitumor efficacy of doxorubicin against breast cancer cells [18]. So we speculated that the formyl peptide receptor might be related to the drug resistance of the tumor cells, and the results of our experiments confirmed our speculation. In this study, the formyl peptide receptor was highly expressed in drug-resistant breast cancer tissues. The apoptosis of MCF-7 and MCF-7/ADM with knockdown of FPRL2 were significantly higher than those of control cells in response to Adriamycin. The p-ERK and p-AKT protein levels were decreased after FPRL2 knocked down. The above results indicate that the FPRL2 is involved in at least part of the function of cellular drug resistance through ERK and AKT pathways, consistent with previous reports [19].

This study confirmed that the knockdown of the FPRL2 can reverse the drug resistance of breast cancer cells, thus there is possibility that FPRL2 can be used as a target for adjuvant breast cancer therapy. Since the ligands of the formyl peptide receptor are numerous but of small molecular weight, it is favorable to synthesize small molecule compounds as antagonists or agonists of the formyl peptide receptor for the adjuvant therapy of breast cancer [20].

## Data Availability

The original contributions presented in the study are included in the article/supplementary material, further inquiries can be directed to the corresponding author.

## Footnotes

1
http://www.kmplot.com/

2
https://depmap.org/portal/interactive/

3
http://autodock.scripps.edu/
